# Therapeutic Effects of Noninvasive Technology Modalities on Lower-Limb Motor Function in Spinal Cord Injury: A Systematic Review

**DOI:** 10.1016/j.arrct.2025.100536

**Published:** 2025-10-15

**Authors:** Siti Ainun Marufa, Hung-Yen Chin, Bor-Shing Lin, Hung-Chou Chen, Tsung-Hsun Hsieh, Wei-Lun Lo, Chun-Wei Wu, Yu-Ting Li, Zidni Immanurohmah Lubis, Nurul Aini Rahmawati, Kurnia Putri Utami, Chih-Wei Peng

**Affiliations:** a International PhD Program in Biomedical Engineering, College of Biomedical Engineering, Taipei Medical University, Taipei, Taiwan; b Department of Physical Therapy, Faculty of Health Science, University of Muhammadiyah Malang, Malang, Indonesia; c Department of Obstetrics and Gynecology, Taipei Medical University Hospital, Taipei, Taiwan; d Department of Obstetrics and Gynecology, School of Medicine, College of Medicine, Taipei Medical University, Taipei, Taiwan; e Department of Computer Science and Information Engineering, National Taipei University, New Taipei City, Taiwan; f Department of Physical Medicine and Rehabilitation, School of Medicine, College of Medicine, Taipei Medical University, Taipei, Taiwan; g Department of Physical Medicine and Rehabilitation, Shuang Ho Hospital, Taipei Medical University, New Taipei City, Taiwan; h School of Physical Therapy and Graduate Institute of Rehabilitation Science, College of Medicine, Chang Gung University, Taipei, Taiwan; i Neuroscience Research Center, Chang Gung Memorial Hospital, Taoyuan, Taiwan; j Division of Neurosurgery, Department of Surgery, Shuang Ho Hospital, Taipei Medical University, New Taipei City, Taiwan; k Department of Surgery, School of Medicine, College of Medicine, Taipei Medical University, Taipei, Taiwan; l School of Biomedical Engineering, College of Biomedical Engineering, Taipei Medical University, Taipei, Taiwan; m National Center for Instrumentation Research, National Institutes of Applied Research, Hsinchu, Taiwan; n School of Gerontology and Long-Term Care, College of Nursing, Taipei Medical University, Taipei, Taiwan

**Keywords:** Lower motor function, Noninvasive stimulation, Rehabilitation, Spinal cord injury, Systematic review, Transcranial direct current stimulation, Transcranial magnetic stimulation

## Abstract

**Objectives:**

To systematically evaluate the effects of noninvasive technology modalities, defined as externally applied stimulation or feedback devices, on lower-limb motor outcomes in individuals with spinal cord injury (SCI), addressing gaps in generalizability and classification to support evidence-based rehabilitation strategies.

**Data Sources:**

We systematically searched PubMed, Web of Science, EMBASE, and the Cochrane Library for English-language articles from database inception to 2023 (initial search conducted in April 2024 and updated in January 2025).

**Study Selection:**

We included randomized controlled trials involving adults with SCI that investigated noninvasive technology modalities applied to any body region and reported outcomes related to motor score, muscle performance, or walking ability. Of 2325 records screened, 22 full-text articles were independently evaluated by 2 reviewers, and 11 met the inclusion criteria and were included in the review.

**Data Extraction:**

Two reviewers independently extracted data from eligible studies, with disagreements resolved through consensus with a third reviewer. The risk of bias (RoB) was assessed using the Cochrane RoB 2 tool by 2 reviewers.

**Data Synthesis:**

Across the included studies (224 participants; mean age=44.9 y), interventions typically consisted of 30-minute sessions, 4 times weekly, over 6 weeks. Four studies applied transcranial stimulation, 4 used transspinal stimulation, and 3 targeted muscle stimulation. Outcomes were evaluated in 4 studies for motor score, 7 for muscle performance, and 7 for walking ability. Most interventions, combined with standard rehabilitation, showed improvements across these outcomes, although only a subset demonstrated statistically significant between-group effects. RoB was low in 6 studies, had some concerns in 3, and was high in 2.

**Conclusions:**

Noninvasive modalities appear effective in enhancing lower-limb motor function in individuals with SCI. However, variability in intervention protocols and methodological quality limits the ability to draw definitive conclusions. Further studies should standardize protocols and minimize bias to strengthen the evidence for SCI rehabilitation strategies.

Spinal cord injuries (SCIs) involve damage and significant neural loss within the spinal cord, resulting in detriment to bodily functions.^1^ Traumatic injury, such as motor vehicle collisions, sports injuries, and fall occurrences, is predominant in most SCI causes.^2^ As a result, SCI is one of the most prevalent and severe conditions in clinical practice, affecting around 100 people per million globally.^3^ The SCI imposes a considerable economic burden, impacting both the health care system and societal development. This condition often leads to mobility limitations, reduced physical activity, sensory impairments, and autonomic deficits, all of which can significantly reduce quality of life.^4^^,^^5^

Moreover, incidences of SCI primarily occur at the thoracic level,^6^ and lower-limb paralysis is the most typical symptom because of a thoracic SCI.^7^ Reports on recovery priorities indicate that individuals with SCI highly emphasize regaining lower-extremity function as a key focus.^8^ However, SCI has long been considered incurable, and the recovery process for SCI may not be sufficiently satisfying.^9^ Recent approaches, such as the use of pharmacologic agents, stem cell transplantation, and biomaterial implantation, have demonstrated beneficial effects in animal models. However, translational efforts have not attained promising clinical results.^10^, ^11^, ^12^ Over the past 3 decades, significant progress has been made in both studying and treating the pathophysiology of SCIs. In particular, rehabilitation in posttrauma care has promoted better prognoses for individuals with SCI by mitigating complications and optimizing residual functions. Nevertheless, the complete restoration of impaired motor function remains limited, and modalities that directly target nerve function could be a valuable addition.^13^

Clinically, SCI is categorized as complete (cSCI) or incomplete (iSCI) based on whether neurologic function persists below the injury site. In cSCI, there is a total loss of voluntary motor and sensory function below the lesion, whereas in iSCI, some degree of function is preserved. Since most cases of SCI are iSCI, residual descending corticospinal pathways remain, offering opportunities to enhance recovery.^14^ Strengthening the connectivity of these pathways and promoting neuroplasticity in motor cortex neurons connected to the spinal cord can facilitate the restoration of motor function.^15^^,^^16^ Novel device-driven interventions for long-term neurologic injuries are becoming available because of the convergence of neuro-engineering fields.^17^^,^^18^ These approaches aim to enhance cortical adaptability and corticospinal connectivity, thereby supporting motor recovery. Delivering stimulation to the spinal cord has been shown to modulate spinal circuits and contribute to functional improvements in the injured central nervous system.^19^ Evidence from animal studies demonstrates that direct electrical stimulation of the motor cortex promotes the growth of corticospinal axons and the formation of new synaptic connections within the spinal cord.^20^ Clinical studies have also shown that noninvasive stimulation can modulate neural activity by enhancing or inhibiting excitability.^21^ Together, these findings provide mechanistic evidence that targeted noninvasive stimulation, particularly when applied to the motor cortex, can drive spinal circuit reorganization and neural plasticity to promote motor recovery after SCI.

Repetitive transcranial magnetic stimulation (rTMS) and transcranial direct current stimulation (tDCS) are classified as noninvasive modalities and are widely used to treat nerve tissues. Adjusting parameters during rTMS applications promotes corticomotor excitability through high-frequency (>5 Hz) stimulation and inhibits it through low-frequency (≤1 Hz) stimulation.^22^ Additionally, intermittent theta-burst stimulation (iTBS), a structured form of rTMS, is delivered through high-frequency stimulation totaling 600 pulses within a shorter duration and has been widely used in research and clinical applications.^23^^,^^24^ In parallel, tDCS can modify central nervous system excitability by applying a direct current of approximately 2 mA,^25^ with anodal stimulation enhancing cortical excitability and cathodal stimulation reducing it. Beyond cortical applications, the spinal cord has also become a target for stimulation because of the preservation of motor and sensory pathways below the lesion.^26^ Collectively, these interventions are referred to as noninvasive technology modalities, defined as externally applied interventions that deliver electrical, magnetic, or mechanical stimulation or feedback to modulate neural or neuromuscular function, without requiring surgical implantation.^27^^,^^28^ Evidence across various functions, including hand grip strength, walking, spinal motor neuron protection, and bladder control, further attests to their potential efficacy.^29^^,^^30^

Despite these promising applications, reported outcomes for motor function recovery in individuals with SCI remain inconsistent across studies.^31^^,^^32^ Previous systematic reviews have assessed the effects of noninvasive neuromodulation; most have focused narrowly on specific anatomical targets, such as cortical or spinal regions, or on particular device types. In contrast, the clinical application of noninvasive modalities is often influenced by resource availability, institutional protocols, and clinician preference, which can vary widely. A broader synthesis that includes a range of stimulation modalities, regardless of anatomical target, is therefore needed to better reflect real-world rehabilitation practices. Furthermore, existing reviews have not categorized outcomes according to their functional relevance, limiting their applicability in clinical decision-making. By classifying outcomes from fundamental motor improvements (muscle strength and motor scores) to higher-level functional gains (such as walking ability), the present review aims to provide clinicians with more actionable insights. This systematic review synthesizes evidence from randomized controlled trials (RCTs) evaluating the effects of noninvasive technology modalities on lower-limb motor function in individuals with SCI.

## Methods

Following the guidelines in the Cochrane Handbook for Systematic Reviews of Interventions,^33^ a prespecified protocol (registration number: CRD42024505759) was registered with the International Prospective Register of Systematic Reviews. Additionally, the Preferred Reporting Items for Systematic Reviews and Meta-Analyses (PRISMA) standards served as the basis for the description of our review.^34^ The PRISMA 2020 Checklist is provided in supplemental appendix S1 (available online only at http://www.archives-pmr.org/).

### Search strategy

We conducted a comprehensive search of 4 electronic databases: PubMed, Web of Science, Cochrane Library, and EMBASE, from their inception to December 2023. The initial search was performed between March and April 2024, and a manual update was conducted to identify relevant articles published in 2024 and early 2025. However, no additional studies meeting the inclusion criteria were identified.

The search was restricted to English-language full-text articles. A senior reviewer (U.I.) supervised the search process. We used a combination of broad keywords and Medical Subject Headings terms to maximize sensitivity in identifying RCTs. Core search terms included “noninvasive stimulation,” “transcranial direct current stimulation,” “transcranial magnetic stimulation,” and “spinal cord injury.” The search syntax used in PubMed was as follows: (noninvasive stimulation [title/abstract]) OR (transcranial magnetic stimulation [title/abstract]) OR (transcranial direct current stimulation [title/abstract]) OR (transcutaneous stimulation [title/abstract]) OR (transspinal stimulation [title/abstract]) OR (neuromodulation technique [title/abstract]) AND (spinal cord injury [title/abstract]). Additionally, we screened the reference lists of relevant systematic reviews to identify any additional eligible studies. The complete search strategies for each database are detailed in supplemental appendix S2 (available online only at http://www.archives-pmr.org/).

### Eligibility criteria

Studies were included in this systematic review if they met the following criteria, defined according to the Participants, Interventions, Comparators, Outcomes, and Study Design framework: (1) population: adults aged 18 years or older with a diagnosis of SCI, confirmed using a recognized neurologic classification, such as the American Spinal Injury Association Impairment Scale (AIS). Both cSCI and iSCI injuries were eligible. While the registered protocol specified chronic SCI as being ≥6 months postinjury, studies that enrolled participants at earlier stages postinjury were also considered, provided all other criteria were satisfied. Participants with unstable medical conditions, including severe infection, advanced cardiovascular or pulmonary disease, or pressure ulcers, were excluded. (2) Intervention: studies that evaluated noninvasive technology modalities. For this review, the term encompassed externally applied device-based interventions, such as rTMS, iTBS, tDCS, transcutaneous or transspinal stimulation, and functional electrical stimulation (FES), as well as robotic-assisted interventions incorporating feedback systems. Both single- and multisession protocols were eligible. (3) Comparator: acceptable comparators included sham stimulation, standard rehabilitation care, or alternative noninvasive technology modalities distinct from the experimental condition. (4) outcomes: studies were required to report at least 1 outcome related to lower-limb motor function, including motor score, muscle performance, or walking ability. Studies were excluded if they did not assess lower-limb motor outcomes or if they exclusively investigated upper-limb or sensory outcomes. (5) Study design: only original, peer-reviewed RCTs published in English with full-text available were included. Case reports, case series, review articles, commentaries, book chapters, conference abstracts, and nonrandomized studies were excluded.

### Study selection and data extraction

Two independent reviewers (S.A.M. and Z.I.L.) screened all records retrieved from the database searches. Duplicates were removed using EndNote (version X9).^a^ Titles and abstracts were screened to exclude irrelevant, unclear, or ineligible studies. Full-text articles were then assessed for eligibility based on the inclusion criteria. Discrepancies during study selection and data extraction were resolved through discussion, and a third reviewer was consulted when consensus could not be reached. A senior reviewer oversaw the entire process.

Data extraction was performed independently by 2 reviewers using a standardized form based on the Cochrane checklist. A structured template was developed to extract relevant data from each included study. Studies were excluded if outcome data were unavailable; reasons for exclusion are listed in supplemental appendix S3 (available online only at http://www.archives-pmr.org/). Extracted variables included: (1) study identifiers (authors and y); (2) study design; (3) participant characteristics (age, sex, and SCI duration, degree, and level); (4) intervention details (modality and electrode placement); and (5) motor function outcomes. Because of the heterogeneity of study designs and outcomes, results were synthesized narratively, highlighting key trends across intervention types, stimulation targets, and motor function outcomes.

### Outcomes

The primary outcomes assessed in this review were lower-limb motor function, categorized into 3 domains: motor score, muscle performance, and walking ability. Motor scores were measured using the AIS and/or lower extremity motor scores (LEMS). Muscle performance was primarily assessed through electromyography (EMG), which provides detailed timing, force, and muscle coordination. Additional muscle function indicators included the root mean square for muscle contraction strength, maximum voluntary contraction (MVC) for peak force output, and the Modified Ashworth Scale (MAS) for evaluating spasticity. Walking ability was evaluated using 1 or more standardized functional tests, including the Holden Walking Ability Scale (HWAS), Walking Index for an SCI, 10-meter walk test (10MWT), 6-minute walk test, Berg Balance Scale, timed Up and Go test, and Spinal Cord Independence Measure (SCIM).

### Risk of bias assessment

The risk of bias (RoB) for each included study was independently assessed by 2 reviewers (N.A.R. and K.P.U.) using the Cochrane RoB 2 tool, in accordance with the Cochrane Handbook for Systematic Review of Interventions. This tool evaluates 5 domains, including the randomization process, deviation from the intended intervention, reporting of missing outcome data, measurement of outcomes, and the possibility of solely reporting intentionally selected data.^35^^,^^36^ A third reviewer was involved to determine the majority decision in the event that approval could not be achieved.

Studies were categorized as having a low RoB, some concerns, or a high RoB based on the Cochrane criteria. A study was rated as low RoB if only 1 domain raised “some concerns.” Ratings of “some concerns” were assigned when 2 or more domains had this classification, and a rating of high RoB was given if any single domain was judged as high risk. Sensitivity analysis was conducted to explore whether the RoB influenced observed intervention effects. Additional attention was given to whether studies had published a prespecified protocol or statistical analysis plan, which informed the assessment of selective reporting bias.

## Results

### Study selection and identification

**Fig 1:**
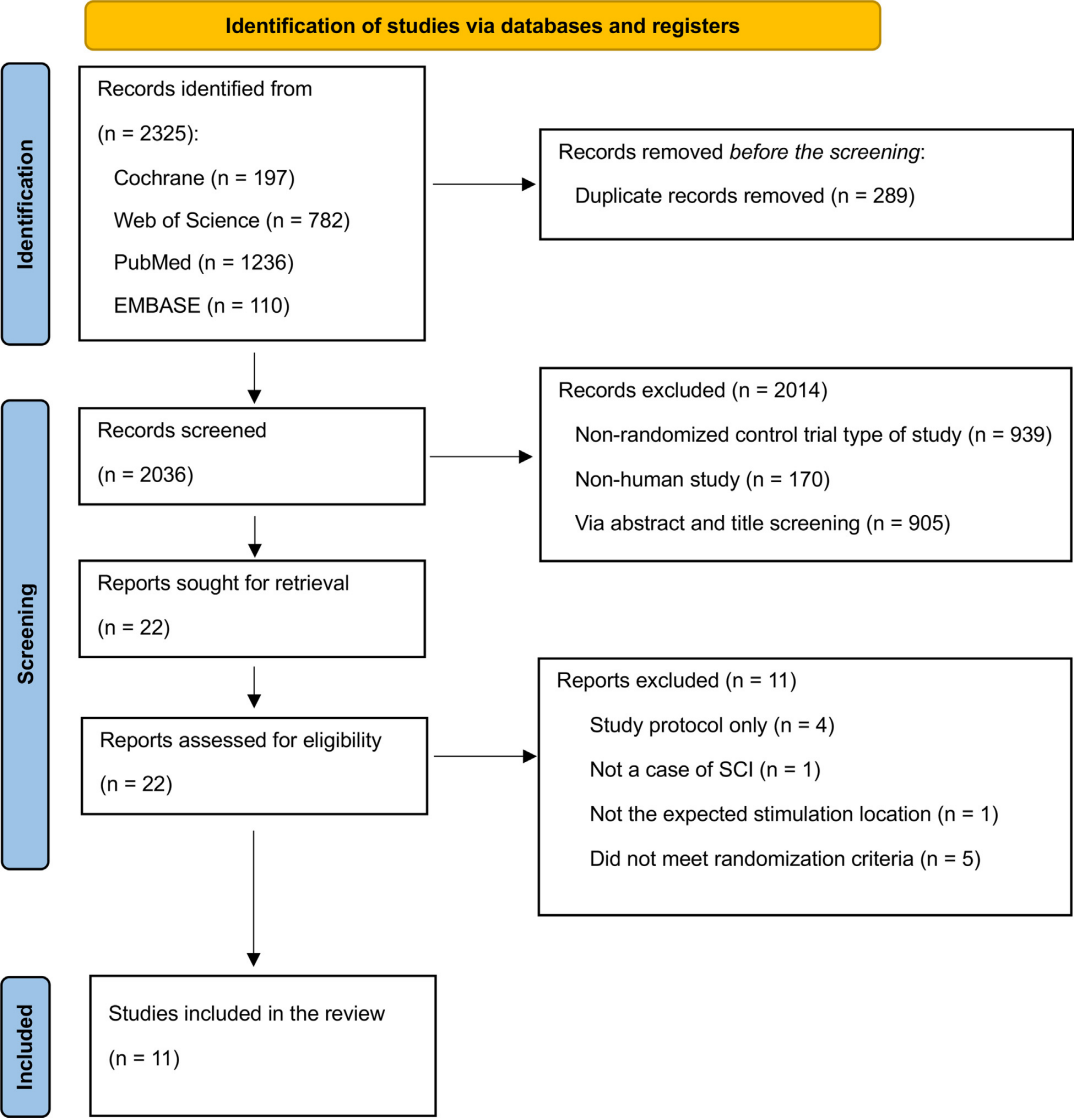
Flowchart of study identification based on the PRISMA statement.

Figure 1 presents the PRISMA flowchart of study selection and identification according to the systematic review. The database search initially yielded 2325 records. After removing 289 duplicates, 2036 records remained for screening. Of these, 2014 were excluded based on title and abstract review, primarily because of study design, population, or intervention type. The full texts of 22 articles were assessed for eligibility, and 11 were excluded for reasons such as inappropriate intervention type, study setting, or population.

Finally, 11 studies met the inclusion criteria and were included in the qualitative synthesis (fig 1). All included studies were published in English. Six trials were conducted in the United States,^37^, ^38^, ^39^, ^40^, ^41^, ^42^ while the others were conducted in China,^43^ Hong Kong,^44^ Denmark,^45^ Brazil,^46^ and India.^47^

### Study description

Table 1 presents the characteristics of the included RCTs, including study setting, design, and participant demographics. Of the 11 studies, 7 employed a parallel design, and 4 used a crossover design. Blinding varied: 5 were double-blind, 5 were single-blind, and 1 did not specify the blinding status. Randomization methods were clearly described in 8 studies, with 6 using computer-generated sequences and 2 employing manual randomization methods. The remaining 3 studies did not provide sufficient details about the randomization.

**Table 1:** Characteristics of studies investigating noninvasive technology modalities for lower-limb motor dysfunction after SCI.

Author, Year (Country)	Study Design	Sample			Other Characteristics		
		N (M/W)	Age (y), Mean ± SD	SCI Type (iSCI/cSCI)	Onset Postinjury, Mean ± SD	Injury Level (C/T/L or as reported)	**AIS Grade Distribution** **(A/B/C/D)**
Powell et al, 2018 (USA)^37^	Randomized, crossover, single-blinded, sham-controlled	6 (4/2)	45.8±14 .0	6/0	188.5±146.3 mo	1/3/2	0/0/4/2
Cheung et al, 2019 (Hong Kong)^44^	Randomized, double-blinded	Exp: 8 (7/1) Con: 8 (4/4)	Exp: 55.6±5.0 Con: 53.0±12.9	Exp: 8/0 Con: 8/0	Exp: 17.0±7.0 mo Con: 10.4±6.3 mo	Exp: C1-L2 Con: C3-L2 (range reported)	Exp: 0/0/7/1 Con: 0/0/4/4
Chou et al, 2020 (USA)^38^	Pseudorandomized, single-blinded	Exp: 10 (9/1) Con: 15 (14/1)	Exp: 30.0±6.3 Con: 28.0±6.2	Exp: 4/6 Con: 5/10	Exp: 16±2.3 mo Con: 13±3.0 mo	Exp: 7/3 Con: 6/9 (tetraplegia/paraplegia)	Exp: 6/-/4 (B-C)/0 Con: 10/-/5 (B-C)/0
Simis et al, 2021 (Brazil)^46^	Randomized, double-blinded, sham-controlled	Exp: 21 (18/3) Con: 22 (15/7)	Exp median (range): 31 (22-42) Con median (range): 41 (32-53)	Exp: 21/0 Con: 22/0	Exp median (IQR): 16 (6-24) mo Con median (IQR): 15.5 (7-23) mo	Exp: 4/17 (tetraplegia/paraplegia) Con: 11/11 (tetraplegia/paraplegia)	Exp: 0/0/10/11 Con: 0/0/10/12
Krogh et al, 2022 (Denmark)^45^	Randomized, single-blinded, sham-controlled	Exp: 10 (8/2) Con: 9 (7/2)	Exp: 57.1±8.3 Con: 51.8±12.1	Exp: 9/1 Con: 9/0	Exp: 91.3±40.8 d Con: 87.3±69.0 d	Exp: 5/4/1 Con: 6/1/2	Exp: 1/0/3/6 Con: 0/0/2/7
Lin et al, 2022 (USA)^39^	Randomized, crossover, sham-controlled	Exp: 7 (4/3) Con: 8 (1/7)	Exp: 52.0±12.6 Con: 41.8±13.3	15/0	Exp: 138.9±80.8 mo Con: 89.3±97.0 mo	Exp: 6/1/0 Con: 6/2/0	Exp: 0/0/2/5 Con: 0/0/1/7
Evans et al, 2022 (USA)^40^	Randomized, double-blinded, sham-controlled	Exp: 11 (8/3) Con: 14 (10/4)	Exp: 50.5±10.7 Con: 46.7±15.0	Exp: 11/0 Con: 14/0	Exp: 78.5±93.0 mo Con: 93.2±85.3 mo	Exp: 9/2/0 Con: 13/1/0	Exp: 0/0/1/10 Con: 0/0/1/13
Pulverenti et al, 2022 (USA)^41^	RCT, single-blinded	Exp: 5 (4/1) Con: 6 (5/1)	45.81±15.47	Exp: 4/1 Con: 5/1	Exp: 8.2±4.4 mo Con: 7.3±4.7 mo	Exp: 3/2/0 Con: 4/2/0	Exp: 1/0/2/2 Con: 1/0/3/2
Hawkins et al, 2022 (USA)^42^	Randomized, crossover of a single intervention, followed by a parallel arm study	Exp: 4 (NR) Con: 4 (NR)	Exp: 52.8±13.2 Con: 51.3±14.1	8/0	Exp: 39.3±22.2 mo Con: 165.5±216.8 mo	Exp: 3/1/0 Con: 2/2/0	Exp: 0/0/1/3 Con: 0/0/0/4
Feng et al, 2023 (China)43	Pilot randomized, double-blinded, sham-controlled	Exp: 19 (15/4) Con: 19 (15/4)	Exp: 45.6±15.3 Con: 37.5±14.2	Exp: 19/0 Con: 19/0	Exp: 109.8±100.8 d Con: 101.1±67.5 d	Exp: 10/3/6 Con: 8/5/6	Exp: D (C-D) median (95% CI) Con: D (C-D) median (95% CI)
Goel et al, 2023 (India)^47^	RCT, parallel, single-centered, participant-blinded	Exp: 9 (7/2) Con: 9 (8/1)	Exp median (95% CI): 41.89 (36.1-47.7) Con median (95% CI): 39.11 (30.1-48.2)	Exp: 9/0 Con: 9/0	Exp median (95% CI): 6.89 (5.8-8.0) mo Con median (95% CI): 7.56 (6.4-8.7) mo	NR	NR

NOTE. “Age” and “Onset Postinjury” data are presented as mean ± SD unless otherwise specified.

Abbreviations: AIS Grade A, complete; AIS Grade B, sensory incomplete; AIS Grade C, motor incomplete, with more than half of key muscles below a neurological level <3/5; AIS Grade D, motor incomplete, with at least half of key muscles below a neurological level ≥3/5; CI, confidence interval; Con, control group; Exp, experimental group; Injury level, number of participants with cervical (C), thoracic (T), or lumbar (L) injuries, unless otherwise specified (eg, tetraplegia/paraplegia or ranges); M, men;. NR, not reported; W, women.

Across the included studies, sample sizes ranged from 4 to 43 participants (mean ± SD, 20±11.6), yielding a total of 224 participants. Sex distribution was reported in most studies, with 163 men (mean ± SD, 16.3±9.9 per study) and 53 women (mean ± SD, 5.3±3.2 per study); 1 study (n=8) did not provide sex data.^42^ The mean age ± SD of participants in the experimental group was 46.2±9.0 years, and in the control group, it was 43.6±7.7 years. Regarding injury chronicity, 9 studies included participants more than 6 months postinjury,^37^, ^38^, ^39^, ^40^, ^41^, ^42^^,^^44^^,^^46^^,^^47^ whereas 2 studies enrolled individuals with an onset of less than 6 months, but were retained as they fulfilled all other eligibility criteria.^43^^,^^45^ Injury level distribution was reported in 7 studies,^37^^,^^39^, ^40^, ^41^, ^42^, ^43^^,^^45^ comprising 76 cervical, 29 thoracic, and 17 lumbar patients; 3 additional studies reported only broad ranges (C1-L2) or tetraplegia versus paraplegia classifications,^38^^,^^44^^,^^46^ and 1 study did not specify the injury level.^47^ According to the AIS classification, 3 studies included participants with cSCI (n=19),^38^^,^^41^^,^^45^ while 8 studies enrolled only individuals with iSCI (n=205).^37^^,^^39^^,^^40^^,^^42^, ^43^, ^44^^,^^46^^,^^47^

Table 2 details the intervention protocols. All experimental groups received noninvasive technology modalities, whereas control groups received sham stimulation (n=7),^37^^,^^39^^,^^40^^,^^42^^,^^43^^,^^45^^,^^46^ standard care (n=2),^38^^,^^44^ or an alternative treatment (n=2).^41^^,^^47^ The types of stimulation delivered to the experimental groups varied: 1 study used iTBS,^43^ 1 used Lokomat (Hocoma) combined with EMG biofeedback,^44^ 2 used FES,^38^^,^^47^ 2 used transcranial magnetic stimulation (TMS),^41^^,^^45^ 2 used tDCS,^40^^,^^46^ and 3 used transspinal direct current stimulation (tsDCS).^37^^,^^39^^,^^42^ Four studies paired stimulation with standard care,^43^^,^^44^^,^^46^^,^^47^ 4 with locomotor training,^40^, ^41^, ^42^^,^^45^ and 3 used stimulation alone.^37^, ^38^, ^39^ Because of differences in study design, the first postintervention assessment was used as the primary follow-up time point. The intervention dosage averaged 24.3 (SD, 22.0) sessions across studies, with a mean ± SD session duration of 25.3±11.8 minutes. When expressed as total intervention time (number of sessions×session duration), interventions provided an average ± SD of 691.8±870.2 minutes over a mean ± SD period of 6.9±6.9 weeks. Despite this variability, most protocols typically consisted of 30-minute sessions, delivered 4 times per week, for 6 weeks.

**Table 2:** Intervention parameters used in selected studies on noninvasive technology modalities after SCI.

Author, Year	Target Site	Group Allocation and Parameters		Standard Care/Rehabilitation	**Total Sessions**
		Exp (FITT)	Control (FITT)		
Powell et al, 2018^37^	Thoracic spinal cord of T10	F=3 sessions (crossover, ≥1 wk washout); I=2.5 mA; T=20 min (ramped 30 s at onset/offset, delivered in 2×10 min blocks); type: anodal/cathodal tsDCS	F=3 sessions (crossover, ≥1 wk washout); I=ramped 2.5 mA for 30 s, then 0 mA for the remaining 8.5 min; T=20 min total (same ramp up/down as Exp); type: sham tsDCS	NR	3
Cheung et al, 2019^44^	Bilateral vastus lateralis	F=3×/wk for 8 wk; I=40% BWS, assist-as-needed; EMG biofeedback (<30% activation threshold); T=30 min/session; type: RABWSTT with EMG biofeedback	F=3×/wk for 8 wk; I=passive only (active-passive exercise); T=30 min/session; type: passive lower-limb mobilization	Mobilization, strengthening, trunk stabilization, wheelchair training, and overground walking	24
Chou et al, 2020^38^	Lower-limb (leg muscle)	F=3×/wk for 26 wk; I=70%-85% max heart rate (progressed from 60%, work:rest=2:1); T=30-40 min/session (6×5 min rowing); type: hybrid-FES rowing	Con A: F=NR; I=NR (tested by graded arms-only exercise); T=NR; type: arms-only ergometer exercise. Con B (waitlist): no structured exercise program	No additional standardized physiotherapy	Exp=78; Con A=NR; Con B=0
Simis et al, 2021^46^	Primary motor cortex	F=3×/wk for 10 wk or 5×/wk for 6 wk; I=2 mA, ramped 30 s; T=20 min/session (10 min each hemisphere, alternating daily); type: anodal tDCS	F=same as Exp; I=2 mA ramped 30 s then off; T=20 min total (30 s active); type: sham tDCS	Lokomat robotic-assisted gait training, progressive protocol; participants also continue usual rehabilitation	30
Krogh et al, 2022^45^	Leg motor cortex	F=5×/wk for 4 wk; I=20 Hz, 100% RMT, 45 trains×40 pulses) T=∼22 min; type: rTMS	F=same as Exp; I=same parameters, coil disconnected, masking with secondary coil; T=∼22 min/session; type: sham rTMS	Lower-limb resistance training and lower-limb physiotherapy training; additional usual care (hydrotherapy, occupational therapy, and activity daily living training)	20
Lin et al, 2022^39^	Thoracic spinal cord of T10-T12	F=single session (crossover, 1 wk apart); I=2.5 mA, ramped 30 s, 10 min constant; T=10 min during treadmill walking with pelvis perturbation; type: anodal tsDCS	F=single session (crossover); I=2.5 mA ramped 10 s then off; T=10 min treadmill walking with pelvis perturbation; type: sham tsDCS	NR	2
Evans et al, 2022^40^	Bilateral motor cortex-cerebellum	F=1/d for 3 d; I=2 mA, ramped 40 s; T=20 min/session during MST; type: anodal tDCS	F=1/d for 3 d; I=2 mA ramped up 40 s then off; T=20 min total; type: sham tDCS	MST with 6 locomotor tasks	3
Pulverenti et al, 2022^41^	Motor cortex (TMS) + thoracolumbar spinal cord (transspinal)	F=5×/wk for ∼5 wk; I=TMS at MEP threshold (double-cone coil)+transspinal at TEP threshold, 240 paired stimuli/session; T=1 h/session (40 min PAS, 20 min stepping); type: PAS (TMS to transspinal)	F=same as Exp; I=same intensities, ISI adjusted, transspinal preceded TMS; T=1 h/session; type: PAS (transspinal to TMS)	Lokomat-assisted locomotor training, treadmill speed, BWS, and leg guidance adjusted	∼26
Hawkins et al, 2022^42^	Thoracic spinal cord (T11-T12)	F=4×/wk for 4 wk; I=2.5 mA, ramped 30 s; T=30 min/session during locomotor training; type: anodal tsDCS	F=4×/wk for 4 wk; I=2.5 mA ramped up 30 s then off; T=30 min/session; type: sham tsDCS	Locomotor training: treadmill and overground stepping	16
Feng et al, 2023^43^	Bilateral motor cortex (leg area)	F=5×/wk for 9 wk; I=100% RMT, 600 pulses; T=∼6 min 40 s/session; type: iTBS	F=5×/wk for 9 wk; I=dummy coil (auditory/tactile cues, no field); T=∼6 min 40 s/session; type: sham iTBS	Multidisciplinary SCI rehabilitation program: motor training, electroacupuncture, biofeedback, robotic lower-limb training, routine rehabilitation, and nursing	45
Goel T et al, 2023^47^	Erector spinae and rectus abdominis	F=5×/wk for 4 wk; I=3 Hz warm-up and recovery, 18 Hz work, visible contraction; T=40 min FES; type: FES	F=5×/wk for 4 wk; I=VR immersive trunk-based games; T=45 min; type: VR	Conventional physical therapy: ROM, mat exercise (rolling, long sitting, kneeling)	20

Abbreviations: BWS, body weight support; Exp, experimental group (defined here as the group receiving noninvasive technology modalities, regardless of how designated in the original study); FITT, frequency, intensity, time, and type; ISI, intenstimulus interval; MEP, motor evoked potential; MST, motor skill training; NR, not reported; PAS, paired associative stimulation; RABWSTT, robot-assisted body weight supported treadmill training; RMT, resting motor threshold; ROM, range of motion; Standard care/rehabilitation, rehabilitation activities provided equally to both groups, if reported; TEP, transspinal evoked potential; VR, virtual reality.

### RoB

**Fig 2:**
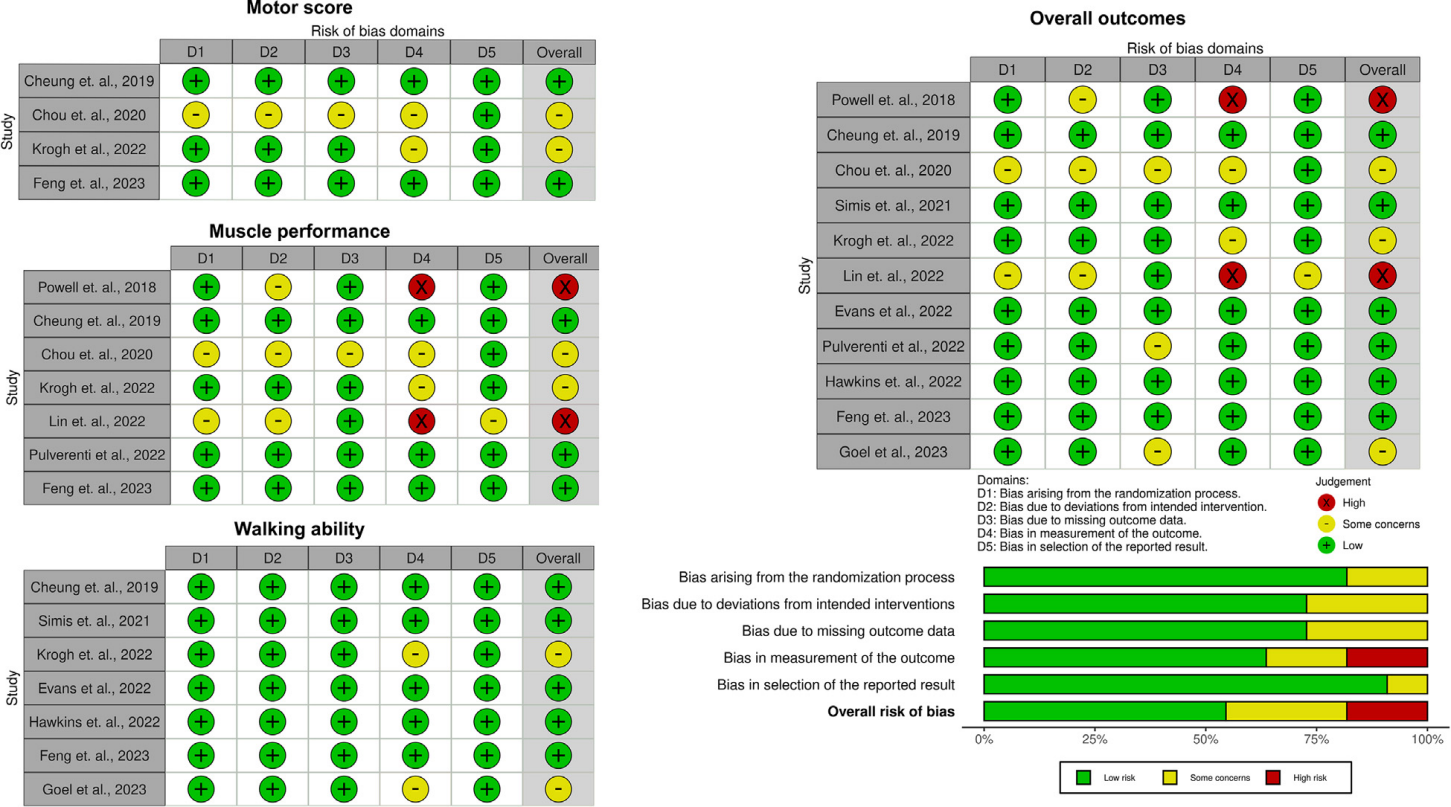
RoB assessment across the included studies using the Cochrane RoB 2 tools. This figure summarizes the RoB assessments for the included studies, categorized into low risk, some concerns, or high risk, across 5 domains (D1-5). Results reflect the overall judgment of each study, with color-coded classifications (green: low risk, yellow: some concerns, red: high risk).

Figure 2 illustrates the distribution of RoB assessments across the 11 included studies, evaluated using the Cochrane RoB 2 tool. Overall, 6 studies were rated as having a low RoB, 3 raised some concerns, and 2 were judged to have a high RoB. Most studies demonstrated low risk across domains, although variability in reporting quality contributed to uncertainty in a subset of trials.

Domain-specific analysis revealed that 82% of studies had a low RoB for the randomization process, while 18% raised some concerns because of insufficient reporting of allocation methods. In the domain of deviations from intended interventions, 73% of studies were rated as low RoB, with the remaining 27% showing some concerns, primarily because of unclear blinding of participants and providers. Similarly, 73% of studies were judged to have a low RoB for missing outcome data, with concerns arising in cases where data were unavailable and potentially related to the outcome. In the measurement of outcome domain, 64% of studies had a low RoB, while 18% raised some concerns, and 18% were rated as high RoB; these ratings were mainly attributed to inadequate reporting of blinding of outcome assessors or potential bias in outcome evaluation. For selective outcome reporting, 91% of studies were judged as low RoB, and 9% raised some concerns because of a lack of access to a published protocol or analysis plan. Overall, most studies demonstrated a low RoB across domains, though variability in reporting quality contributed to uncertainty in a subset of trials.

### Outcomes of the interventions

#### Motor score

Motor function below the level of injury is often severely impaired in individuals with SCI, and restoring lower-limb mobility is a core rehabilitation goal.^8^ Among the 11 included studies, 4 reported outcomes using LEMS to quantify changes in motor strength.^38^^,^^43^, ^44^, ^45^ As summarized in table 3, iTBS combined with physical therapy over 9 weeks significantly improved LEMS compared with the sham group (*P*<.0001).^43^ Lokomat with EMG biofeedback also showed significant within-group improvement (*P*=.043), although not when compared with the control group (*P*=.061).^44^ A study assessing hybrid-FES rowing for 6 months found no significant differences compared with standard care (*P*=.28).^38^ In contrast, 20 sessions of rTMS resulted in greater LEMS improvement at discharge compared with sham stimulation (*P*=.014).^45^ Overall, while some interventions showed promising effects on lower-limb motor scores, the findings varied in significance and consistency across trials, highlighting the need for further research with standardized protocols and longer follow-up periods.

**Table 3:** Outcomes measured and findings of noninvasive technology modalities studies for SCI.

Author, Year	Outcomes Measured	Findings
Powell et al, 2018^37^	Muscle performance: MEP	No significant within-group changes for anodal (*P*=.70), cathodal (*P*=.09), or sham tsDCS (*P*=.82). No significant between-group differences among anodal, cathodal, and sham tsDCS (all *P*≥.10).
Cheung et al, 2019^44^	Motor score: LEMS Muscle performance: L-force Walking ability: WISCI II	LEMS improved significantly within groups (*P*=.043), but showed no between-group difference (*P*=.326). L-force showed no significant within-group or between-group changes (all *P*>.05). WISCI II improved significantly within groups (*P*=.003), but there was no overall between-group effect (*P*=.382); a significant time×group interaction was detected (*P*=.02).
Chou et al, 2020^38^	Motor score: AIS Muscle performance: MAS	Within-group increases in the AIS motor score were observed in both groups, but there were no significant between-group differences (mean difference=1.3, 95% CI, −19.9 to 4.4, *P*=.53). MAS showed no significant within- or between-group differences (mean difference=6.4, 95% CI, −4.2 to 17, all *P*>.05).
Simis et al, 2021^46^	Walking ability: WISCI II	No significant between-group difference after whole sessions (*P*=.45). At posttreatment, a significantly greater proportion of the experimental group improved compared with the control group (*P*=.046). The effect was maintained at follow-up (*P*=.046). Logistic regression confirmed significance at posttreatment (*P*=.013) and follow-up (*P*=.014).
Krogh et al, 2022^45^	Motor score: LEMS Muscle performance: MVC Walking ability: 10MWT	LEMS improved significantly within the experimental group (*P*<.01) but not in the control group (*P*=.22), with a significant between-group difference favoring the experimental group (*P*<.02). MVC increased more in the experimental group than the control group, but no significant main effects were found (treatment: *P*>.15; treatment×time: *P*>.76). In the 10MWT, both groups showed significant within-group improvements (experimental group: *P*<.01; control group: *P*<.02), but there were no significant between-group differences (*P*=.16).
Lin et al, 2022^39^	Muscle performance: EMG	Muscle activity was significantly greater with anodal tsDCS compared with sham during late adaptation (Abd: *P*=.030; RF: *P*=.045; VM: *P*=.041), early postadaptation (Abd: *P*=.046; MH: *P*=.044), and late postadaptation (Abd: *P*=.034). Other muscle differences were not significant (all *P*>.05).
Evans et al, 2022^40^	Walking ability: 10MWT	Walking speed increased significantly within both MST + tDCS (*P*<.01) and MST + sham groups (*P*<.01). No significant between-group differences were observed. Mean change (0.13 m/s) was approached but did not reach the MCID of 0.15 m/s.
Pulverenti et al, 2022^41^	Muscle performance: EMG	In the TMS to transspinal condition, early reflex showed phase-dependent modulation (*P*=.004), but no time effect was observed (*P*=.185), while late reflex showed no significant changes (*P*>.05). In the transspinal to TMS condition, TA short-latency reflex amplitude was significantly reduced (*P*=.021), as well as long-latency reflex (*P*<.001), with no change in reflex gain or threshold (*P*>.05).
Hawkins et al, 2022^42^	Walking ability: 10MWT	Walking speed increased in the tsDCS + LT group (mean change ± SD, 0.18±0.29 m/s), with 3 of 4 participants exceeding the MCID (0.06 m/s). In the sham + LT group, the average ± SD change was −0.05±0.23 m/s, with 1 of 4 participants exceeding MCID. No between-group *P* value was reported.
Feng et al, 2023^43^	Motor score: LEMS Muscle performance: RMS Walking ability: HWAS	LEMS improved significantly in both iTBS and sham groups (*P*<.001), with greater improvement in the iTBS group (group×time: *P*<.0001). RMS of the quadriceps femoris showed significant within-group changes (*P*=.001) and greater gains in the iTBS group for both left (*P*=.010) and right sides (*P*=.0101). HWAS also improved significantly within groups (*P*=.002), with greater gains in the iTBS group (group×time: *P*=.0003).
Goel et al, 2023^47^	Walking ability: SCIM	FES + CPT showed significant within-group improvements (*P*=.01). Between groups, VR + CPT produced greater gains in self-care (*P*=.006), mobility (*P*=.004), and total scores (*P*=.006), with no difference in respiration management (*P*=.53). Median total score change (8 points) did not exceed the MCID.

Abbreviations: Abd, abductor; CI, confidence interval; CPT, conventional physical therapy; L-force, load force; MCID, minimal clinically important difference; MEP, motor evoked potential; MH, medial hamstring; MST, motor skill training; RF, rectus femoris; RMS, root mean square; TA, tibialis anterior; VM, vastus medialis; VR, virtual reality; WISCI II, Walking Index for Spinal Cord Injury version II.

#### Muscle performance

Seven studies assessed lower-limb muscle performance using a range of physiological measures. Five studies used EMG,^37^^,^^39^^,^^41^^,^^43^^,^^44^ while 2 other studies used MAS^38^ and MVC.^45^ As shown in table 3, iTBS was associated with significant increases in muscle activity relative to sham stimulation (*P*<.0011).^43^ One tsDCS study reported increased EMG activity in the hip abductors (*P*<.05),^39^ though another using a similar protocol found no significant EMG changes (*P*=.09).^37^ Transspinal-TMS applied to the thoracolumbar level significantly enhanced flexion reflex amplitudes (*P*<.021).^41^ Lokomat with EMG biofeedback did not significantly alter load force values (*P*=.112), nor were there significant group differences (*P*=.121).^44^ In a study using rTMS, MVC values showed moderate improvement in leg strength (effect size=0.40), although no significant main effects were found (time: *P*>.23; treatment: *P*>.15; group and time interaction: *P*>.76).^45^ MAS-based assessment in a hybrid-FES group revealed no significant changes in spasticity compared with standard care (*P*>.05).^38^ These mixed results suggest modality-specific effects on muscle performance that warrant further exploration.

#### Walking ability

Regaining walking capability is crucial for individuals with SCI, significantly improving their quality of life.^48^, ^49^, ^50^ Walking ability was assessed using validated clinical measures; one study used HWAS, which can integrate detailed components of gait, balance, and mobility.^43^ Two studies applied the Walking Index for an SCI II to specifically evaluate walking performance.^44^^,^^46^ Three other studies used the 10MWT, which involves timing and measuring the walking speed of participants.^40^^,^^42^^,^^45^ One study used SCIM to assess mobility, including walking performance, in individuals with SCI.^47^

As detailed in table 3, iTBS significantly improved HWAS scores compared with the sham group (*P*=.0003).^43^ Lokomat with EMG biofeedback demonstrated significant group-time interaction results (*P*=.02).^44^ Similarly, rTMS with usual care reduced 10MWT scores (*P*<.01),^45^ while tDCS combined with Lokomat training showed no significant between-group difference at 15 days (*P*=.45), but significant improvements were observed at posttreatment and follow-up (both *P*=.046).^46^ Other tDCS studies also demonstrated within-group improvements in walking speed and distance (*P*<.001), although between-group differences were not statistically significant.^40^ tsDCS combined with locomotor training was associated with improvements in walking speed, with most participants exceeding the minimal clinically important difference, although no between-group *P* value was reported.^42^ FES interventions improved SCIM mobility scores significantly (*P*<.01).^47^ Together, these findings suggest that several noninvasive technology modalities, particularly when combined with task-specific rehabilitation, can enhance lower-limb functional ambulation in individuals with SCI.

## Discussion

This review examined the therapeutic effects of noninvasive technology modalities on motor score, muscle performance, and walking ability in individuals with SCI. The main findings for each functional domain are discussed below.

### Effects on motor score

Building on the promising potential of noninvasive intervention to enhance motor recovery after SCI, our analysis of motor score outcomes across the reviewed studies supports these therapeutic effects. While there is some variability in the results, several studies reported significant improvements in LEMS. This suggests that noninvasive modalities have positively and consistently improved motor scores after SCI. Two studies using iTBS and rTMS demonstrated relatively larger improvements.^43^^,^^45^ Both interventions involved high-frequency stimulation directed at the primary motor cortex, which is associated with the leg. Consistent with earlier studies, 15 days of rTMS delivery at 20 Hz to the vertex, combined with gait training, significantly enhanced lower-limb motor strength in individuals with iSCI.^51^ Similarly, administering real rTMS at 20 Hz over 20 sessions before initiating gait rehabilitation yielded greater improvements in motor strength compared with the sham stimulation.^52^ These studies suggest that repeated activation of corticospinal pathways through excitatory stimulation may promote synaptic plasticity and strengthen residual motor circuits. Of note, the rTMS trial primarily enrolled individuals with iSCI; 1 participant with cSCI was also included in the experimental arm.^45^ Likewise, one trial investigated hybrid-FES rowing in a mixed cohort of iSCI and cSCI but did not demonstrate significant motor gains compared with virtual reality-combined standard care.^38^ From a mechanistic standpoint, including both iSCI and cSCI participants allows investigation of spinal circuit responsiveness under conditions of minimal descending input.^53^ However, interpretation of functional motor recovery in cSCI remains limited and should be distinguished from findings in iSCI. Hybrid-FES rowing may still benefit subsets of patients, particularly in early rehabilitation, by improving cardiometabolic health and mitigating the risk of cardiovascular disease.^54^^,^^55^ These results highlight the distinction between interventions designed to improve motor strength versus those aimed at enhancing systemic health or functional performance.

In contrast, interventions targeting peripheral systems yielded more variable results.^38^^,^^44^ We noted that one included study combined Lokomat-assisted gait training with EMG biofeedback, which differs from stimulation-based modalities such as tDCS or rTMS. Its inclusion was consistent with our predefined eligibility criteria, as it represents a device-based intervention providing neuromuscular feedback. This trial reported within-group improvements in LEMS but no significant between-group effect. The intervention primarily targeted walking ability, with robotic assistance supporting gait, while EMG biofeedback facilitated voluntary muscle activation, leading to improvements in gait symmetry, particularly through the normalization of hip and knee joint movements during the swing phase.^44^ This may explain why gains in isolated muscle strength were not observed. Although distinct from direct stimulation techniques, this feedback-based robotic approach highlights a complementary avenue for promoting motor recovery after SCI. Taken together, cortical stimulation techniques showed more consistent effects on motor score improvements, likely because of their ability to modulate central motor pathways.

### Effects on muscle performance

Seven of the 11 studies reported outcomes related to lower-limb muscle performance using varied assessment tools.^37^, ^38^, ^39^^,^^41^^,^^43^, ^44^, ^45^ The results predominantly indicated positive effects on muscle performance after noninvasive interventions. Muscle performance improvements can occur through 2 mechanisms: recruiting a greater number of motor units and enhancing the activation frequency of those already engaged.^43^ High-frequency iTBS and rTMS have been shown to enhance motor cortex excitability, which indirectly facilitates greater motor unit recruitment, leading to stronger muscle contractions.^56^ Furthermore, these TMS parameters can increase the firing rate of activated motor units, thereby enhancing both the force and precision of muscle movement. Complementing these findings, the results of a transspinal-TMS Paired Associative Stimulation (PAS) suggest specific modulation of spinal reflex pathways.^41^ The observed significant change in short-latency flexion reflex amplitude indicates heightened excitability in spinal motor circuits, potentially supporting improved neuromuscular coordination. This trial^41^ also included participants with cSCI; its findings are better understood as evidence of how stimulation can modulate spinal reflex pathways under conditions of absent or minimal voluntary descending input, rather than as evidence of improved voluntary muscle activation or strength in cSCI.

Additionally, 2 single session crossover studies using tsDCS reported inconsistent effects on muscle performance.^37^^,^^39^ One found no significant changes in motor evoked potential (MEP) amplitudes within or between groups, which may be because of the absence of MEPs, small sample sizes, or suboptimal parameters.^37^ The other^39^ showed time-dependent increases in EMG activity across multiple muscles, indicating polarity-dependent modulation of spinal excitability that may enhance neuromuscular recruitment during walking.^57^ Such effects can outlast stimulation, suggesting a potential for sustained muscle activation.^58^ In contrast, the hybrid-FES rowing intervention studied,^38^ which also included participants with cSCI, did not demonstrate significant between-group effects on spasticity as measured by MAS. These results further emphasize the limited evidence available for muscle performance outcomes in cSCI, and their interpretation should remain cautious.

### Effects on walking ability

One of the primary rehabilitation priorities for individuals with iSCI is improving functional walking ability.^59^ Mobility skills, including walking distance and speed, are key indicators of walking capacity and are strong predictors of independence after SCI.^60^ Seven studies in this review assessed walking ability using various assessment tools.^40^^,^^42^, ^43^, ^44^, ^45^, ^46^, ^47^ Across these studies, most interventions combining noninvasive technology modalities with locomotor training or physical therapy yielded improvements in walking outcomes, although the strength of evidence varied. Between-group effects were consistently observed in cortical stimulation approaches, particularly in iTBS and tDCS, with the latter also demonstrating sustained gains at follow-up.^43^ FES intervention also improved mobility; however, in one trial, conventional training enhanced with virtual reality led to significantly greater improvements in the mobility domain of SCIM, encompassing walking-related activities, compared with FES.^38^ Yet, the median total score change did not exceed the established minimal clinically important difference, suggesting that the differences may not reflect a clinically meaningful improvement. In contrast, spinal stimulation approaches, such as tsDCS, produced clinically relevant gains in walking speed, with most participants reaching the threshold of clinical importance, although statistical confirmation was limited by the small sample size.^42^ Overall, these findings indicate that different modalities have the potential to enhance walking recovery after SCI, while the current evidence base remains heterogeneous and further validation is needed.

Improvements in walking ability were most apparent when stimulation was combined with locomotor training, supporting the concept that neuromodulation is most effective when paired with repetitive, task-specific practice.^61^ This synergy arises because locomotor training, as an activity-based therapy, induces activity-dependent plasticity that reorganizes spinal networks and strengthens synaptic connectivity,^62^ while stimulation lowers activation thresholds and enhances the responsiveness of locomotor circuits to residual descending input and sensory feedback.^63^^,^^64^ Such mechanisms are thought to enhance the efficiency of muscle synergies and motor coordination, which can manifest as improvements in step length, gait symmetry, balance, and walking endurance, ultimately supporting greater functional mobility.^65^ The tDCS combined with locomotor training has shown benefits that extend beyond the intervention, indicating durable neuroplastic adaptations. Similarly, tsDCS trials suggest clinically meaningful improvements in walking speed. Overall, these findings support evidence that pairing stimulation with activity-based therapies enhances both immediate and longer-term locomotor recovery.^66^, ^67^, ^68^ Clinically, noninvasive modalities should be considered as adjuncts, not replacements, for locomotor training to maximize sustained improvements in walking after SCI.

### Implications for research and practice

This review highlights the therapeutic potential of noninvasive technology modalities targeting different sites for lower-limb motor recovery in SCI, reflecting real-world rehabilitation settings where the clinical application of noninvasive modalities is often shaped by institutional protocols, resource availability, and clinical preference. The inclusion of diverse modalities, regardless of anatomical target, offers a more comprehensive perspective that aligns with a varied clinical environment. While several studies reported positive effects, heterogeneity in study parameters produced inconsistent findings. Small sample sizes, variability in demographics, injury characteristics, and adjunct therapies further influenced outcomes. Older adults, a substantial subgroup of individuals with SCI, generally show reduced recovery potential.^69^ These limitations underscore the need for future studies to use standardized protocols and stratified participant selection to enhance the quality of evidence. Such efforts would strengthen the development of personalized rehabilitation approaches using noninvasive technology modalities for individuals with SCI.

### Study limitations

This review has several limitations that should be considered when interpreting the findings. First, there was substantial heterogeneity across the included trials. The intervention dose varied widely, ranging from single session to multiweek protocols, intended to evaluate longer-term outcomes. Session frequency and duration were also inconsistent, which likely contributed to variability in reported effects and limited comparability across studies. Second, participant chronicity was inconsistently reported, with a few trials enrolling individuals <6 months postinjury, despite our protocol specifying chronic SCI. To address this, we reported chronicity using <6 months versus ≥6 months categories, but such variability remained a source of heterogeneity. Third, a small number of trials included participants with cSCI alongside predominantly iSCI cohorts. While such inclusion is mechanistically valuable for probing spinal circuit responsiveness under minimal descending input, the evidence base of this review primarily reflects iSCI, and generalizability to complete injuries is limited. Fourth, the reporting of participant characteristics, such as sex, age, and injury level, was incomplete in several studies, though these factors are known to influence treatment response. Fifth, outcome reporting was not standardized, and cointerventions varied, further complicating cross-study comparisons. Sixth, although we expanded our search strategy, relevant studies may have been missed because of indexing limitations, inconsistent terminology, or keyword restrictions. Finally, this review did not systematically assess adverse effects or tolerability, which are critical for evaluating the safety and clinical feasibility of noninvasive technology modalities.

### Conclusions

This review highlights the potential of noninvasive technology modalities targeting the brain, spinal cord, or peripheral musculature as adjuncts to standard care in SCI. Reviewed benefits include improvements in motor scores, muscle performance, and walking ability, suggesting support for functional recovery when appropriately applied. By integrating outcomes across diverse stimulation targets and functional domains, this synthesis offers a clinically relevant framework for rehabilitation. However, methodological heterogeneity and small sample sizes limit the strength of the current evidence. Future studies should optimize stimulation parameters, assess long-term effects, and recruit larger, stratified cohorts to improve the generalizability and advance personalized rehabilitation strategies for individuals with SCI. From a clinical perspective, these modalities should be regarded as complementary tools that can enhance, but not replace, conventional rehabilitation, with the greatest benefit expected when paired with structured locomotor or task-specific training.

## Supplier

a. EndNote, version X9; Clarivate.

## Disclosure

The investigators have no financial or nonfinancial disclosures to make in relation to this project.

## Appendix Supplementary materials

Image, application 1

Image, application 2

Image, application 3

## Footnotes

Supplementary material associated with this article can be found, in the online version, at doi:10.1016/j.arrct.2025.100536.
