# Supplementary material for: Therapeutic Effects of Noninvasive Technology Modalities on Lower-Limb Motor Function in Spinal Cord Injury: A Systematic Review
Source: Arch Rehabil Res Clin Transl. 2025 Oct 15;7(4):100536. doi: 10.1016/j.arrct.2025.100536 (PMC12750423; doi:10.1016/j.arrct.2025.100536)
Supplement: Supplementary file 2 [file mmc2.docx]

**Appendix B. Electronic search strategy**

| **Database** | **Search** | | **Filters** | | **Quantity** | | |
| --- | --- | --- | --- | --- | --- | --- | --- |
| Web of Science  (topic) | ((((((TS=(non-invasive stimulation)) OR TS=(transcranial magnetic stimulation)) OR TS=(transcranial direct current stimulation)) OR TS=(transcutaneous stimulation)) OR TS=(transpinal stimulation)) OR TS=(neuromodulation technique)) AND TS=(spinal cord injury) | | Articles, controlled-clinical trial, randomized-controlled trial, human study. | | 782 | | |
| PubMed  (title, abstract, free full text) | ((((((non-invasive stimulation) OR (transcranial magnetic stimulation)) OR (transcranial direct current stimulation)) OR (transcutaneous stimulation)) OR (transspinal stimulation)) OR (neuromodulation technique)) AND (spinal cord injury) | | Controlled-clinical trial, randomized-controlled trial, human study | | 1236 | | |
| Cochrane  (title, abstract, keywords) | #1 | MeSH descriptor: [Transcranial Direct Current Stimulation] explode all trees | None | 197 | | |  |
|  | #2 | MeSH descriptor: [Transcranial Magnetic Stimulation] explode all trees |  |  |  |  |  |
|  | #3 | MeSH descriptor: [Electric Stimulation Therapy] explode all trees |  |  |  |  |  |
|  | #4 | #1 OR #2 OR #3 |  |  |  |  |  |
|  | #5 | MeSH descriptor: [Spinal Cord Injuries] explode all trees |  |  |  |  |  |
|  | #6 | #4 AND #5 |  |  |  |  |  |
| EMBASE | ('non-invasive stimulation' OR 'transcranial magnetic stimulation' OR 'transcranial direct current stimulation' OR 'transcutaneous stimulation' OR 'neuromodulation' AND 'spinal cord injury'):ti,ab,kw AND ([Controlled Clinical Trial]/lim OR [Randomized Controlled Trial]/lim) | | None | | 110 |  |  |
| Total |  | |  | | 2325 |  |  |
