# Supplementary material for: Therapeutic Effects of Noninvasive Technology Modalities on Lower-Limb Motor Function in Spinal Cord Injury: A Systematic Review
Source: Arch Rehabil Res Clin Transl. 2025 Oct 15;7(4):100536. doi: 10.1016/j.arrct.2025.100536 (PMC12750423; doi:10.1016/j.arrct.2025.100536)
Supplement: Supplementary file 3 [file mmc3.docx]

**Supplementary File 3 Table 3. Reasons for exclusion**

| **Study** | **Title** | **Reason** |
| --- | --- | --- |
| Abualait & Ibrahim | Spinal direct current stimulation with locomotor training in chronic spinal cord injury | Case study, not a randomized-control trial (RCT) |
| Gorgey & Khalil | Neuromuscular Electrical Stimulation Training Increases Intermuscular Fascial Length but Not Tendon Cross-Sectional Area After Spinal Cord Injury | Stimulation target is not relevant to study focus |
| Knikou & Murray | Repeated transspinal stimulation decreases soleus H-reflex excitability and restores spinal inhibition in human spinal cord injury | Quasi-experimental design, did not meet RCT criteria |
| Krenn et al. | Bipolar transcutaneous spinal stimulation evokes short-latency reflex responses in human lower limbs alike standard unipolar electrode configuration | Study focuses on healthy subjects, not spinal cord injury (SCI) cases |
| Leister et al. | The effect of extracorporeal shock wave therapy in acute traumatic spinal cord injury on motor and sensory function within 6 months post-injury: a study protocol for a two-arm three-stage adaptive, prospective, multi-center, randomized, blinded, placebo-controlled clinical trial | Study protocol only  No completed trial data |
| Mao et al. | Effects of cortical intermittent theta burst stimulation combined with precise root stimulation on motor function after spinal cord injury: a case series study | Case series study, did not meet RCT criteria |
| Seanez | Enhanced selectivity of transcutaneous spinal cord stimulation by multielectrode configuration | Crossover study design, did not meet RCT criteria |
| Shackleton et al. | Motor and autonomic concomitant health improvements with neuromodulation and exercise (MACHINE) training: a randomised controlled trial in individuals with spinal cord injury | Study protocol only  No completed trial data |
| Siu et al. | Novel Noninvasive Spinal Neuromodulation Strategy Facilitates Recovery of Stepping after Motor Complete Paraplegia | Case study, did not meet RCT criteria |
| Skiadopoulos et al. | Priming locomotor training with transspinal stimulation in people with spinal cord injury: study protocol of a randomized clinical trial | Study protocol only  No completed trial data |
| Sun et al. | Effects of paired associative magnetic stimulation between nerve root and cortex on motor function of lower limbs after spinal cord injury: study protocol for a randomized controlled trial | Study protocol only  No completed trial data |
